# Changes in legal referrals to specialty substance use disorder treatment from 2015–2019

**DOI:** 10.1186/s40352-024-00297-2

**Published:** 2024-11-06

**Authors:** Carrie E. Fry, Jacob Harris, Marguerite E. Burns

**Affiliations:** 1 Department of Health Policy, Vanderbilt University School of Medicine, 2525 West End Avenue, Suite 1275-G, Nashville, TN 37203 USA; 2 College of Arts and Letters, University of Notre Dame, Notre Dame, IN USA; 3 Department of Population Health Sciences, UW-Madison School of Medicine and Public Health, Madison, WI USA

**Keywords:** Substance use disorder, Structural determinants of health, Legal system

## Abstract

**Background:**

The policy landscape around substance use has changed dramatically in the past decade, which may have affected the number and characteristics of treatment episodes for substance use disorder (SUD). In this study, we examine changes in the volume of SUD treatment referrals from the legal system and compare changes in the composition of substances used by referral source. We used publicly available discharge data on specialty SUD treatment episodes in the U.S. from 2015–2019 and included episodes involving adults that are discharged from specialty SUD treatment facilities during the study. We calculated descriptive statistics of specialty SUD treatment discharges in each year and aggregated across all years by referral source and substance(s) reported upon admission. To test differences by year and referral source, we conducted z-tests of proportions.

**Results:**

The proportion of referrals to specialty SUD treatment from the legal system declined between 2015 and 2019 (*p* < 0.001). However, referrals from probation/parole and diversionary programs grew over time (*p* < 0.001) in number and proportion over time. Legal referrals were most often associated with alcohol or cannabis use, though referrals for these substances declined from 2015–2019.

**Conclusions:**

This research lays the groundwork for future investigations to evaluate the effect of important policy changes on referral sources to specialty SUD treatment and the quality and outcomes associated with referrals to treatment from the legal system.

## Background

Over the past two decades, the U.S.’s approach to substance use substantially changed. The criminalization of substance use beginning in the 1980s led to the incarceration of 1 in every 198 Americans with more than 12.2% for drug-related offenses (Franco, 2010). Incarceration did not reduce recidivism or criminalized behavior associated with substance use as many policymakers had anticipated, (Franco, 2010), and recent incarceration increases the risk of overdose substantially compared to community-dwelling peers (Finlay et al., 2022). U.S. federal and state governments have implemented many policies to reduce the risk of criminal legal interactions related to substance use in the past 10 years, including legalization of illicit substances, the introduction of problem-solving courts, and changes to the organization and delivery of healthcare services for people recently incarcerated.

Taken together, these health and legal system policy changes likely have effects on the number and kinds of referrals to specialty SUD treatment. Prior analyses of referrals to specialty SUD treatment have generally focused on subpopulations, defined by gender (Lucabeche & Quinn, 2022), age (Kopak & Smith-Ruiz, 2016), or mental health status. Prior analyses of referral to specialty SUD treatment via the legal system use relatively outdated data from 2008–2010 (Bronson et al., 2017; Fearn et al., 2016; Smith & Strashny, 2016). These analyses focused on characteristics of SUD and treatment patterns among incarcerated people (Bronson et al., 2017) and people on probation/parole (Fearn et al., 2016). There has been little research on changes in referral patterns to specialty SUD treatment among a general population of people with SUD since the advent of these policies. Such research would provide empirical evidence on the responsiveness of the treatment system to external policies. This study characterizes referrals to specialty SUD treatment between 2015 and 2019 by from legal- and non-legal referral sources and substance use upon admission to inform future research directions of referrals to and quality of specialty SUD treatment.

## Data & Methods

We conducted a retrospective, cross-sectional analysis from 2015–2019 using the publicly available Treatment Episode Dataset – Discharges (TEDS-D) from the Substance Abuse and Mental Health Services Administration (SAMHSA). TEDS-D is a deidentified episode-level dataset collected by states from specialty SUD treatment providers and includes patient and episode characteristics. We excluded 2014 because discharges in 2014 may represent admissions in 2013, prior to the implementation of the ACA’s coverage provisions. We excluded data from 2020 due to the disruptive nature of the COVID-19 pandemic on both the healthcare and legal system. We excluded five states (AK, GA, HI, OR, WV) that did not provide data during the study period (2015–2019). We included all episodes involving adults (18 + years of age) reported to TEDS-D during the study period, as this is the group subject to most of the policies changes of interest. From 2015–2019, TEDS-D captured 8.8 million referrals to specialty SUD treatment that meet our inclusion and exclusion criteria.

TEDS-D captures the following referral sources to specialty SUD treatment: patients/family/friends; other community referrals [defined as community or religious organizations; any federal, state, or local agency that provides aid in areas of poverty relief, unemployment, shelter, or social welfare; defense attorneys; and self-help groups like alcoholics anonymous or narcotics anonymous]; another healthcare provider, including other SUD treatment providers; an employer or school; and the legal system. Within the legal system, there are five possible referral sources: 1) probation/parole; 2) state or federal court (including the formal adjudication process); 3) other legal entities (e.g., local law enforcement agency, corrections agency, youth services, or a review board/agency); 4) diversion programs (including Treatment Alternatives to Street Crime [TASC] programs, driving under the influence/driving while intoxicated [DUI/DWI] programs); and 5) prison.

In addition to referral source, patients can report up to three substances upon admission – a primary, secondary, and tertiary substance used. We included all substances reported upon admission, regardless of position. We categorized substances in the following way: alcohol, cannabis, heroin, cocaine, other opioids (including methadone, synthetic opioids, and prescription opioid pain relievers), benzodiazepines, and other stimulants (including amphetamines and methamphetamines).

We calculated annual counts and proportions of treatment episodes across all referral sources and then by specific legal sources. For proportions of discharges by overall referral source, we used all discharges in TEDS-D in the year of interest as the denominator. For proportions of discharges by specific legal referral sources, we used all legal-system-referred discharges in the year of interest as the denominator. We then conducted z-statistics to determine if the proportion of referrals from each source changed between 2015 and 2019. We also compared substance use upon admission by referral source – categorizing the referrals source as either a non-legal or legal source (including all 5 types). We also conducted z-test statistics by substance reported upon admission by kind of referral source.

## Results

**Fig. 1:**
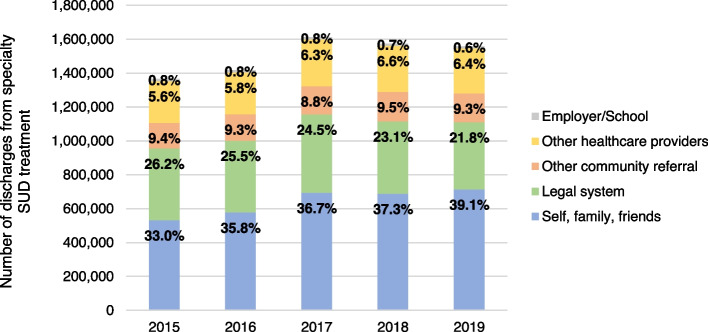
Number and proportion of specialty substance use treatment by referral source, 2015–2019. Source/Notes: SOURCE Authors’ analysis of TEDS-D data between 2015–2019; NOTES Data includes all specialty SUD treatment discharges among adults 18 + years of age. Alaska, Georgia, Hawaii, Oregon, and West Virginia do not consistently report data for TEDS-D and are thus omitted from these analyses

Referrals to specialty SUD treatment from the legal system made up 24.2% of all 8.3 M treatment episodes between 2015–2019, with only the self/family/friend category of referrals making up a larger proportion (36.4%; Fig. 1). The number and proportion of referrals from the legal system to SUD treatment declined by more than 25,000 episodes or 4.4 percentage points (26.2 vs. 21.8%; *p* < 0.001) between 2015–2019. Conversely, the number and proportion of referrals from patients and their family or friends increased by approximately 109,000 episodes or 6.1 percentage points from 2015 to 2019 (33.0 vs. 39.1%; *p* < 0.001). Other sources, including healthcare providers, schools/employers, and community referral sources represented a consistently small proportion of referrals to specialty SUD treatment.

**Fig. 2:**
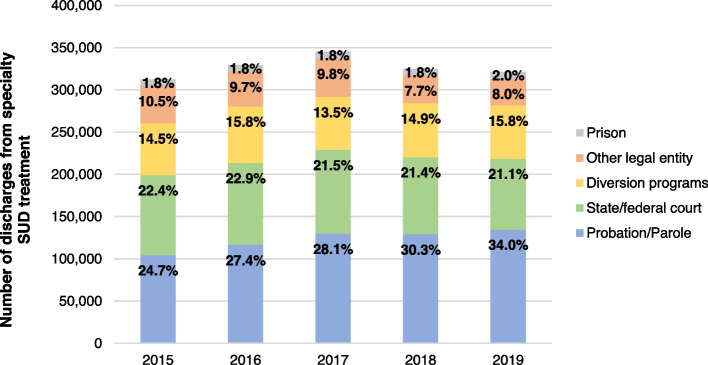
Number and proportion of specialty substance use treatment by legal system referral source, 2015–2019. Source/Notes: SOURCE Authors’ analysis of TEDS-D data between 2015–2019; NOTES Data includes all specialty SUD treatment discharges among adults 18 + years of age. Alaska, Georgia, Hawaii, Oregon, and West Virginia do not consistently report data for TEDS-D and are thus omitted from these analyses

Within the legal system, adults referred to treatment from probation or parole comprised the largest identified source across all years — averaging 28.8% of legal-referred episodes (Fig. 2). Referrals from probation or parole increased by 9.2 percentage points from 2015–2019 (24.7 vs. 34.0%; *p* < 0.001), the largest growth across all referral sources. Diversion programs made up the second largest source of legal system referrals, accounting for 15% of all legal system referrals between 2015–2019.

### Substances reported upon admission

**Fig. 3:**
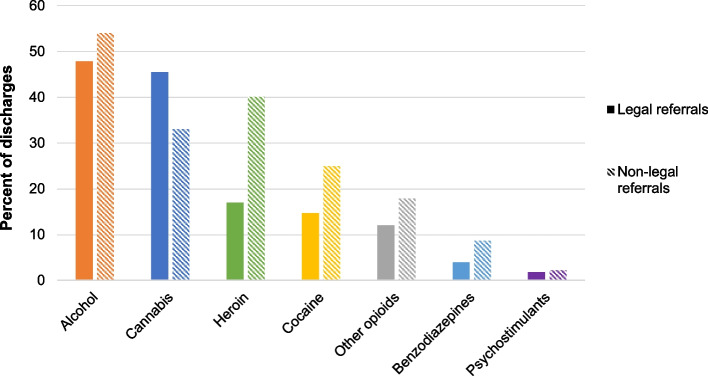
Percent of specialty substance use treatment discharges with reported use of drug by referral source, 2015–2019. Source/Notes: SOURCE Authors’ analysis of TEDS-D data between 2015–2019; NOTES Data includes all specialty SUD treatment discharges among adults 18 + years of age. Alaska, Georgia, Hawaii, Oregon, and West Virginia do not consistently report data for TEDS-D and are thus omitted from these analyses

**Fig. 4:**
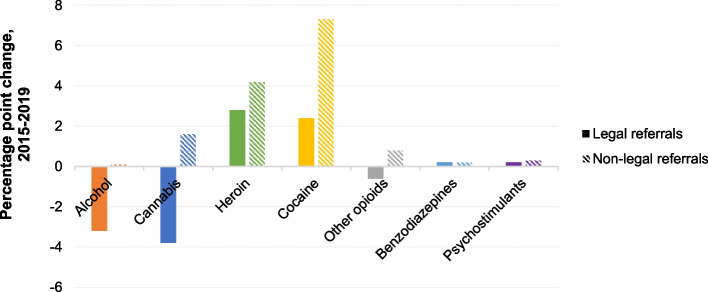
Percentage point change in proportion of discharges by reported drug use and referral source, 2015 – 2019. Source/Notes: SOURCE Authors’ analysis of TEDS-D data between 2015–2019; NOTES Data includes all specialty SUD treatment discharges among adults 18 + years of age. Alaska, Georgia, Hawaii, Oregon, and West Virginia do not consistently report data for TEDS-D and are thus omitted from these analyses

Alcohol was present in approximately half of all treatment episodes, regardless of referral source (47.8% of legal referrals, 54.0% of non-legal referrals; *p* < 0.001) (Fig. 3). From 2015–2019, the proportion of episodes with reported alcohol use remained relatively stable among non-legal referrals (a change of less than 0.1 percentage points), but legal referrals for alcohol declined by 3.2 percentage points (Fig. 4). Cannabis was implicated in almost half of all referrals from legal sources but only a third of non-legal sources (45.5 vs. 33.1%; *p* < 0.001). However, the proportion of legal-system-referred episodes for cannabis declined by 3.8 percentage points from 2015–2019 while the proportion of cannabis-related episodes from non-legal referrals increased by 1.6 percentage points.

The percentage of episodes with heroin implicated was much higher among non-legal referral sources compared to episodes from legal referral sources (40.1% vs. 17.0%; p < 0.001) (Fig. 3). Between 2015–2019, the proportion of heroin-involved episodes referred from non-legal sources grew by 1.4 percentage points more than legal system referrals (4.2 vs. 2.8; Fig. 4). Reported use of other opioids was relatively stable over time, regardless of referral source, and comprised 18.0% of treatment episodes from non-legal referral sources and 12.0% of treatment episodes from legal referral sources (*p* < 0.001; Fig. 3).

One quarter of episodes originating from non-legal referral sources involved cocaine, while 14.7% of legal system referrals involved cocaine (*p* < 0.001). The proportion of non-legal episodes that included cocaine grew approximately three times faster than the proportion of legal referrals for cocaine (7.3 vs. 2.4 percentage points) between 2015–2019. Finally, episodes with reported other stimulant use made up 1.8% of all legal system referrals and 2.3% of all non-legal system referrals (*p* < 0.001), but referrals for stimulant use from either kind of source changed little.

### Limitations

The study data and analyses have limitations. We cannot observe any measure of judicial discretion or treatment system capacity to understand why some people are referred to treatment while others are not. While states are supposed to report data on specialty SUD treatment to SAMHSA, our analytic dataset does not include all states. Additionally, the categorization of legal system entities comes directly from the TEDS-D dataset, leading to ambiguous or overlapping sources of referral like the separation of “defense attorneys” from “state/federal courts”. Our results are descriptive in nature and quantify changes over time; we could not attribute these changes to any specific policy or program. Finally, we cannot follow a person through multiple treatment stays to understand patterns of referral sources nor can we comment on the quality or adequacy of treatment received from any referral source.

## Discussion

 The proportion and number of specialty SUD treatment episodes resulting from all legal referral sources declined between 2015–2019, but referrals from probation or parole increased significantly. This study is the first to document recent changes in population-level referral sources to specialty SUD treatment. These changes could be the result of health or justice-related policies implemented during this period. While additional empirical analyses are required to determine causation, this work provides possible avenues for this line of inquiry.

The legalization of previously-illicit substances may be one potential reason why the proportion of treatment referrals from the legal system are in decline. Since 2012, 31 states have decriminalized cannabis possession, and 21 states have legalized recreational cannabis use. The decriminalization of cannabis led to a reduction in cannabis-related offenses in states with decriminalization laws (Farley & Orchowsky, 2019) compared to states without. We found large declines in legal system referrals for cannabis between 2015 and 2019, a time period during which 19 states decriminalized cannabis possession. Over the same time, non-legal system referrals with reported cannabis use increased. This work mirrors other work on cannabis decriminalization (Gunadi & Shi, 2022).

At the same time, the organization and delivery of health care services in the United States changed dramatically for people with SUDs. The implementation of the Affordable Care Act’s (ACA) coverage provisions in 2014 was designed to improve coverage and access to care for Americans with SUD by requiring the majority of insurers to cover treatment services at parity with other benefits (Abraham et al., 2017; Bainbridge, 2012; Busch et al., 2013; Humphreys & Frank, 2014; Watkins et al., 2015). Research has demonstrated that the ACA’s coverage provisions are associated with increasing rates insurance coverage for people with SUD (Andrews et al., 2019; Olfson et al., 2021a, 2021b, 2021c; Olfson et al., 2021a, 2021b, 2021c; Olfson et al., 2021a, 2021b, 2021c; Saloner et al., 2016, 2017), increased use of SUD treatment (Khatri et al., 2021; Saloner & Maclean, 2020; Saloner et al., 2018), and decreased crime rates (He & Barkowski, 2020; Simes & Jahn, 2022; Vogler, 2020) and recidivism (Fry et al., 2020).

Additionally, several bills passed in the 2010s – the Comprehensive Addiction Recovery Act (CARA) of 2016, the 21st Century Cures Act, and the Substance Use Disorder Prevention that Promotes Opioid Recovery and Treatment for Patients and Communities (SUPPORT) Act of 2018 – provided substantial resources for SUD prevention, harm reduction, and treatment. These laws could have a mixed effect on referrals to SUD treatment. Additional resources for prevention could keep people from needing or receiving treatment, resulting in decreased referrals to specialty SUD treatment. While increased resources for treatment could result in more people accessing and receiving treatment from both non-legal and certain legal referral sources. Previous analyses have demonstrated that CARA was associated with increases in the supply of buprenorphine-waivered providers and thus access to treatment, especially in rural areas (Barnett et al., 2019; Lee et al., 2021), though another study demonstrated that many states had failed to spend money appropriated to them via the 21st Century Cures Act (Murrin, 2020). The timing of passage and appropriation of these bills, as well as the varied implementation across states, makes them difficult to evaluate (The Congressional Budget Office, 2022).

The use of problem-solving courts has also increased since the early 2010s with more than 3,000 problem-solving courts in use across the US (Franco, 2010). Problem-solving courts may refer people to treatment as an alternative to incarceration, as a condition of probation/parole, or through a post-adjudication drug court. The growth of problem-solving courts could be driving the increase in number of referrals from diversion programs and probation or parole, potentially signalling an increased emphasis on public health or medical approaches to SUD in these systems. Additional empirical analyses are needed to understand the implications of these shifts toward treatment, the type and quality of treatment provided, and the outcomes associated with treatment provided via legal system referrals.

## Conclusion

This study examines changes in referrals to specialty SUD treatment between 2015 and 2019 and highlights possible avenues for future investigation into the policy mechanisms that drive these changes. A decrease in the proportion of treatment episodes that result from legal system referrals could represent an increase in access to care or decriminalization, evidenced by an increase in self referrals. However, an increase in the number of episodes referred from probation or parole and diversion programs suggests potential improvements in post-release connections to care and problem-solving courts, respectively.

In order to determine whether these shifts in referral source represent net benefits for people with SUD, additional research on the characteristics of people referred to treatment, the kinds of treatment provided via legal system referrals, and the outcomes of treatment episodes that occur legal system referral is necessary. For instance, previous work has demonstrated that people referred to treatment from the legal system were less likely to receive medications for opioid use disorder (Donahoe et al., 2024; Khatri et al., 2021; Winkelman et al., 2020). Specifically, future research may highlight the kinds of treatment or treatment referral sources that are particularly effective at reducing SUD-related harms and future interactions with the legal system.

## Data Availability

Data are public use files that can be downloaded from the U.S. Substance Abuse and Mental Health Services Administration (SAMSHA) and are available from the authors upon reasonable request.

## Abbreviations

SUD: Substance use disorder
ACA: Affordable Care Act
CARA: Comprehensive Addiction Recovery Act
TEDS-D: Treatment Episode Dataset—Discharges
SAMHSA: Substance Abuse and Mental Health Services Administration
SUPPORT: Substance Use Disorder Prevention that Promotes Opioid Recovery and Treatment for Patients and Communities
